# Synthesis of Zr_2_WP_2_O_12_/ZrO_2_ Composites with Adjustable Thermal Expansion

**DOI:** 10.3389/fchem.2017.00105

**Published:** 2017-11-21

**Authors:** Zhiping Zhang, Weikang Sun, Hongfei Liu, Guanhua Xie, Xiaobing Chen, Xianghua Zeng

**Affiliations:** ^1^Department of Electrical and Mechanical Engineering, Guangling College, Yangzhou University, Yangzhou, China; ^2^School of Physiccal Science and Technology, Yangzhou University, Yangzhou, China

**Keywords:** Zr_2_WP_2_O_12_, ZrO_2_, composites, thermal expansion, ceramics

## Abstract

Zr_2_WP_2_O_12_/ZrO_2_ composites were fabricated by solid state reaction with the goal of tailoring the thermal expansion coefficient. XRD, SEM and TMA were used to investigate the composition, microstructure, and thermal expansion behavior of Zr_2_WP_2_O_12_/ZrO_2_ composites with different mass ratio. Relative densities of all the resulting Zr_2_WP_2_O_12_/ZrO_2_ samples were also tested by Archimedes' methods. The obtained Zr_2_WP_2_O_12_/ZrO_2_ composites were comprised of orthorhombic Zr_2_WP_2_O_12_ and monoclinic ZrO_2_. As the increase of the Zr_2_WP_2_O_12_, the relative densities of Zr_2_WP_2_O_12_/ZrO_2_ ceramic composites increased gradually. The coefficient of thermal expansion of the Zr_2_WP_2_O_12_/ZrO_2_ composites can be tailored from 4.1 × 10^−6^ K^−1^ to −3.3 × 10^−6^ K^−1^ by changing the content of Zr_2_WP_2_O_12_. The 2:1 Zr_2_WP_2_O_12_/ZrO_2_ specimen shows close to zero thermal expansion from 25 to 700°C with an average linear thermal expansion coefficient of −0.09 × 10^−6^ K^−1^. These adjustable and near zero expansion ceramic composites will have great potential application in many fields.

## Introduction

Lots of materials known to show positive thermal expansion as temperature increase. In contrast, some materials show completely different thermal expansion properties and contract upon heating. This negative thermal expansion (NTE) phenomena has been found in some A_2_(MO_4_)_3_ compounds, where the A cation can be a trivalent main group metal, transition metal, or rare earth element ranging from Lu to Ho, while M corresponds to W or Mo (Sumithra and Umarji, 2004, 2006; Liu H. F. et al., 2012; Liu Q. Q. et al., 2012; Liu et al., 2015). In addition, compounds with aliovalent cations on the A and M site have been reported. For example, Zr_2_WP_2_O_12_ has been reported to exhibit strong and stable NTE over a wide temperature range. Zr_2_WP_2_O_12_ adopts the orthorhombic Sc_2_W_3_O_12_ structure, which consists of ZrO_6_ octahedra that share corners with two WO_4_ tetrahedra and four PO_4_ tetrahedra. Zr-O-W (P) linkages in this structure will lead to the volume contraction due to transverse vibration of bridging oxygen atoms as temperature increase (Isobe et al., 2008, 2009; Cetinkol and Wilkinson, 2009; Tani et al., 2010).

Thermal expansion is an important property of materials, and mismatch in thermal expansion often induces unstable performance or failure of devices in the field of microelectronics, optics and micromachines. To avoid the above problems, control of thermal expansion of materials can be necessary. An easy approach is to mix the NTE material with the positive thermal expansion material in the right proportion.

Most studies describing attempts to synthesize controllable thermal expansion composites mainly focus on ZrW_2_O_8_ based composites, such as ZrW_2_O_8_/ZrO_2_ (De Buysser et al., 2004; Lommens et al., 2005; Yang et al., 2007; Khazeni et al., 2011; Romao et al., 2015), ZrW_2_O_8_/Cu and ZrW_2_O_8_/polyimide (Yilmaz, 2002; Sullivan and Lukehart, 2005; Yang et al., 2010; Hu et al., 2014). The coefficient of thermal expansion (CTE) of the composites drops with the increase of the ZrW_2_O_8_ filler. However, the cubic NTE phase of ZrW_2_O_8_ is metastable at room temperature, and has to be prepared by rapid quenching after sintering at 1,200°C. Cubic ZrW_2_O_8_ show a isotropic NTE over a wide temperature range, but a phase transition from α -ZrW_2_O_8_ to β -ZrW_2_O_8_ occurs around 160°C, which leads to the decrease of CTE. This change in thermal expansion may be disadvantageous for composite design. Moreover, when heated to 740°C, ZrW_2_O_8_ decomposes into ZrO_2_ and WO_3_ (Mary et al., 1996; Banek et al., 2010; Gao et al., 2016). In addition, cubic ZrW_2_O_8_ undergoes a pressure induced phase transition to an orthorhombic phase with a positive CTE. This transformation has been observed in composites during thermal cycling, and leads to irreproducible thermal expansion behavior (Perottoni and Jornada, 1998; Miao et al., 2004; Varga et al., 2007; Liu et al., 2014).

Zr_2_WP_2_O_12_ is a new NTE material for use as a filler to adjust the CTE of ceramics, glasses, metals, and polymers. It exhibits a strong NTE over the broadest temperature range (room temperature to its sublimation point of about 1,600°C). Moreover, it does not suffer from the same limitations as ZrW_2_O_8_, as it is thermodynamically stable and does not undergo any phase transformations.

The synthesis and NTE behavior of Zr_2_WP_2_O_12_ ceramics have been reported previously (Isobe et al., 2008, 2009; Cetinkol and Wilkinson, 2009; Tani et al., 2010). Zr_2_WP_2_O_12_ ceramics show stable NTE with an average linear CET of about −5 × 10^−6^ K^−1^. In addition, the Zr_2_WP_2_O_12_ ceramics display excellent mechanical properties (Isobe et al., 2008, 2009; Cetinkol and Wilkinson, 2009). ZrO_2_ ceramics and fibers has been widely used in optics, electronics and high temperature fields (Lommens et al., 2005; Yang et al., 2007). In some special occasions, ZrO_2_ need to keep precision dimensional stability with the change in temperature, because a mismatch in size among different precision devices can cause some problems. The average linear CTE of ZrO_2_ is about 10 × 10^−6^ K^−1^ from room temperature to 1,000°C. The absolute values of the CTE of ZrO_2_ and Zr_2_WP_2_O_12_ are thus similar but have opposite signs, suggesting that these materials are good candidates for the preparation of ceramic composites with tunable CTEs. It is beneficial that ZrO_2_ does not react with Zr_2_WP_2_O_12_ at high temperatures, as it is a starting material in the solid state synthesis of Zr_2_WP_2_O_12_.

A new series of Zr_2_WP_2_O_12_/ZrO_2_ ceramic composites that are expected to show an adjustable CTE were synthesized by a solid state reaction method. This work is devoted to exploring the effects of mass ratio of Zr_2_WP_2_O_12_ and ZrO_2_ on the microstructure, density, and CTE values of the Zr_2_WP_2_O_12_/ZrO_2_ ceramic composites.

## Experimental details

All Zr_2_WP_2_O_12_, ZrO_2_, and Zr_2_WP_2_O_12_/ZrO_2_ ceramics (mass ratios: 1:1, 2:1, 3:1, 4:1) were synthesized through a conventional solid state route. The raw materials were ZrO_2_ (Aladdin, purity ≥99.95%), WO_3_ (Aladdin, purity ≥99.95%), and NH_4_H_2_PO_4_ powders (Aladdin, purity ≥99.5%). A summary of samples prepared can be found in Table 1. Reactant mixtures were milled for 6 h to form a homogeneous powder and dried at 80°C, followed by heating at 500°C for 3 h. After this pre-sintering step, the mixtures were uni-axially cold pressed into pellets of 7 mm in diameter and about 2 mm in thickness. Pellets were calcined at 1,200°C in air for 6 h and cooled down in the furnace.

**Table 1:** Synthesis conditions for ZrO_2_/Zr_2_WP_2_O_12_ ceramics.

**Mass ratio of Zr_2_ WP_2_O_12_:ZrO_2_**	**m(ZrO_2_)/g**	**m(WO_3_)/g**	**m(NH_4_H_2_PO_4_)/g**
0:1	10	0	0
1:1	6.99	1.87	1.86
2:1	7.18	2.99	2.97
3:1	6.58	3.36	3.34
4:1	5.18	2.99	2.97
1:0	3.97	3.74	3.71

Powder X-ray diffraction experiments were performed on a Shimadzu XRD 7000 using CuKα radiation. Data were collected at 40 kV and 30 mA over the 10° to 60° 2θ range with a scanning speed of 5°/min. The fractured surface morphologies of the samples were observed using a TESCAN VEGA3 scanning electron microscope (SEM). The relative densities of the resulting samples were measured using Archimedes' method. The CTEs of the samples were measured with a Seiko 6300 TMA/SS thermal mechanical analyzer at a heating rate of 5°C/min in air between 25 and 700°C.

## Results and discussion

### XRD analysis

**Figure 1:**
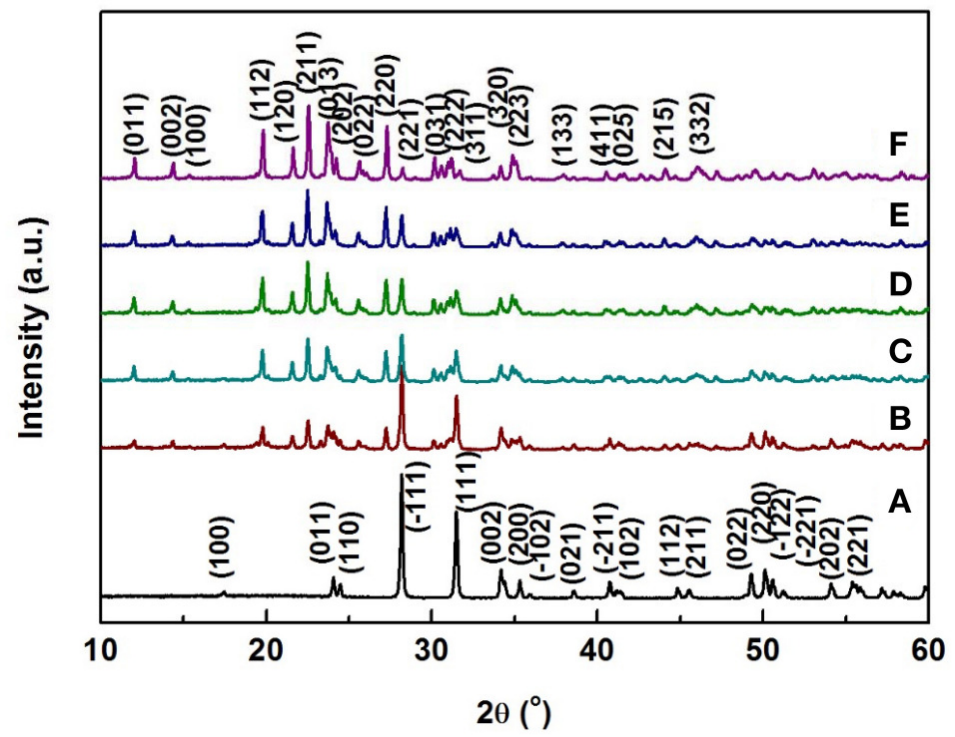
XRD patterns of ZrO_2_, Zr_2_WP_2_O_12_, and Zr_2_WP_2_O_12_-ZrO_2_ composites with different mass ratios sintered at 1,200°C for 6 h. **(A)** ZrO_2_, **(B)** 1:1 Zr_2_WP_2_O_12_:ZrO_2_, **(C)** 2:1 Zr_2_WP_2_O_12_:ZrO_2_, **(D)** 3:1 Zr_2_WP_2_O_12_:ZrO_2_, **(E)** 4:1 Zr_2_WP_2_O_12_-ZrO_2_, **(F)** Zr_2_WP_2_O_12_.

Figure 1 shows typical room temperature XRD patterns of Zr_2_WP_2_O_12_/ZrO_2_ composites with different mass ratios synthesized at 1,200°C for 6 h. The XRD patterns of pure ZrO_2_ and pure Zr_2_WP_2_O_12_ ceramics are also displayed for reference. For pure ZrO_2_ ceramics (Figure 1A), all observed reflections could be well indexed and attributed to monoclinic ZrO_2_ in agreement with JCPDS card number 65–1,023. For pure Zr_2_WP_2_O_12_ ceramics (Figure 1F), all diffraction peaks matched those expected for orthorhombic Zr_2_WP_2_O_12_ (JCPDS 43-0258). No impurity phases were detected. XRD patterns of Zr_2_WP_2_O_12_/ZrO_2_ composites with mass ratios of 1:1, 2:1, 3:1, and 4:1 (Figures 1B–E) displayed diffraction peaks belonging to both monoclinic ZrO_2_ and orthorhombic Zr_2_WP_2_O_12_. As no intermediate phase exists between ZrO_2_ and Zr_2_WP_2_O_12_, no reaction can occur between excess ZrO_2_ and Zr_2_WP_2_O_12_. As expected, the diffraction peaks of Zr_2_WP_2_O_12_ became more intense with increasing mass ratio of Zr_2_WP_2_O_12_.

### SEM and density analysis

**Figure 2:**
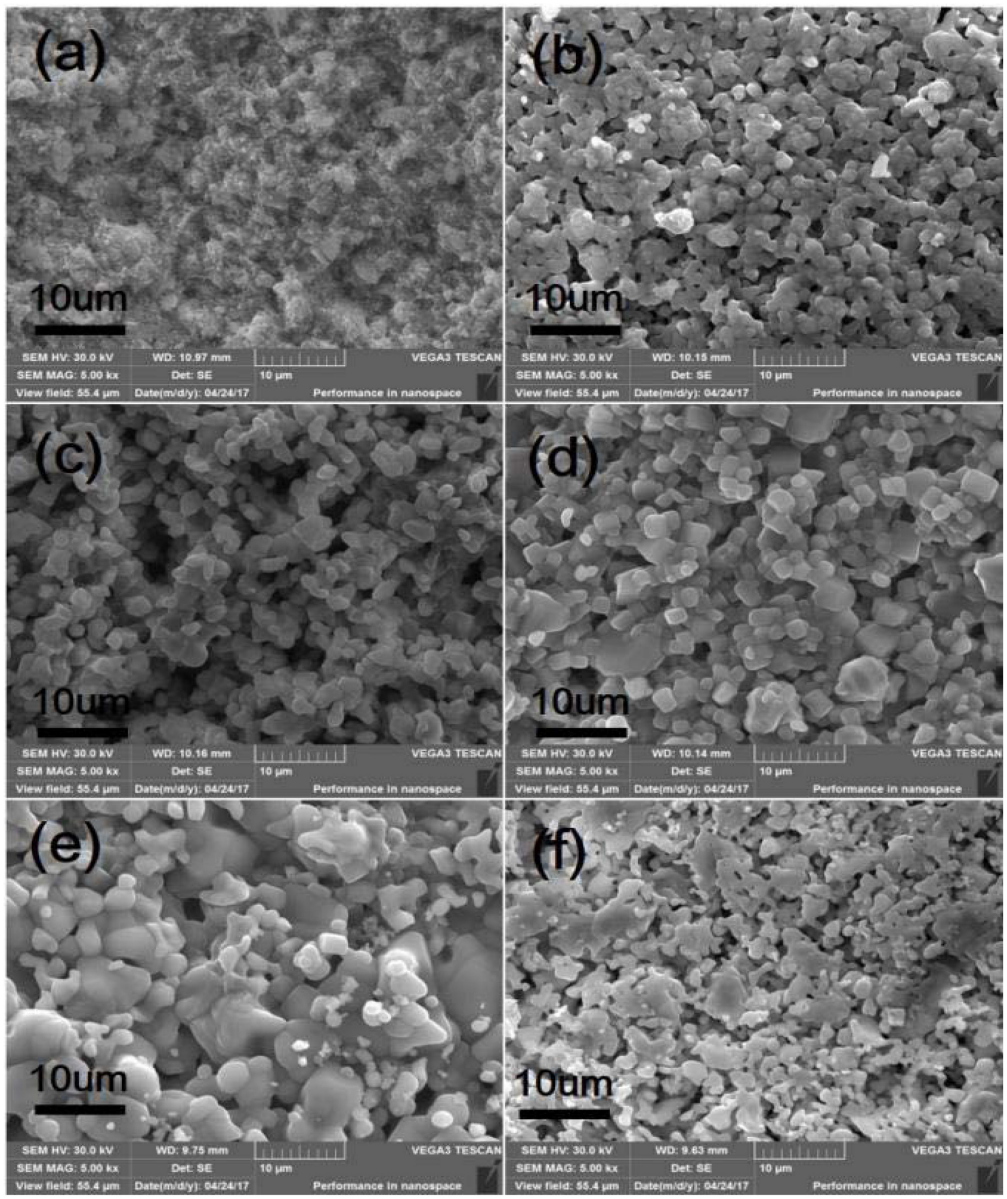
SEM images of ZrO_2_, Zr_2_WP_2_O_12_, and Zr_2_WP_2_O_12_-ZrO_2_ composites with different mass ratios sintered at 1,200°C for 6 h, **(a)** ZrO_2_, **(b)** 1:1 Zr_2_WP_2_O_12_:ZrO_2_, **(c)** 2:1 Zr_2_WP_2_O_12_:ZrO_2_, **(d)** 3:1 Zr_2_WP_2_O_12_:ZrO_2_, **(e)** 4:1 Zr_2_WP_2_O_12_-ZrO_2_, **(f)** Zr_2_WP_2_O_12_.

**Figure 3:**
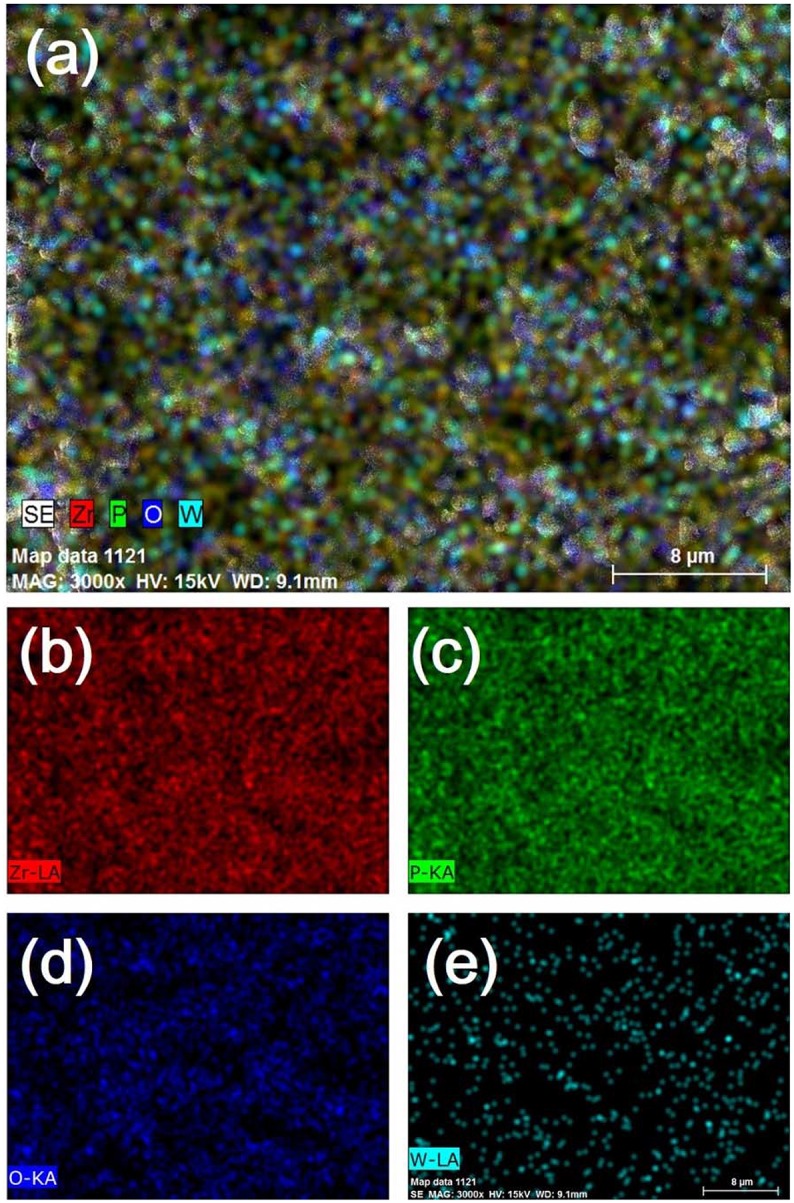
EDX composition maps **(a)** Zr, P, W and O, **(b)** Zr, **(c)** P, **(d)** O, and **(e)** W analysis for 2:1 Zr_2_WP_2_O_12_:ZrO_2_ composite.

SEM micrographs of different weight ratio Zr_2_WP_2_O_12_/ZrO_2_ ceramic composites, ZrO_2_ and Zr_2_WP_2_O_12_ ceramics after sintering at 1,200°C for 6 h are shown in Figure 2. The SEM image of the ZrO_2_ ceramics (Figure 2a revealed significant porosity, which is likely due to insufficient sintering. It is known that the sintering temperature required to fabricate dense and tough ZrO_2_ ceramics is higher than 1,400°C (Varga et al., 2007). Figures 2b–e show SEM images of sintered Zr_2_WP_2_O_12_/ZrO_2_ ceramic composites as a function of different mass ratios. With increasing amount of Zr_2_WP_2_O_12_, Zr_2_WP_2_O_12_/ZrO_2_ ceramic composites sintered for the same time at the same temperature became denser and displayed larger grain sizes and less porosity. The average grain size of 1:1 Zr_2_WP_2_O_12_/ZrO_2_ composites was about 2–3 μm, but increased to about 6–8 μm when the mass ratio of Zr_2_WP_2_O_12_/ZrO_2_ was increased to 4:1. Pure Zr_2_WP_2_O_12_ (Figure 2f) showed a wide size distribution of spherical grains with some residual porosity, which is in agreement with results reported earlier (Isobe et al., 2008, 2009; Cetinkol and Wilkinson, 2009). Figure 3 shows the composition maps analysis of the 2:1 Zr_2_WP_2_O_12_:ZrO_2_ composite. Homogeneous spatial distributions of Zr, P, W, and O elements were observed. These results indicates that Zr_2_WP_2_O_12_ and ZrO_2_ phase uniformly distributed as expected.

In this work, the densities of the resulting Zr_2_WP_2_O_12_, ZrO_2_, and Zr_2_WP_2_O_12_/ZrO_2_ (mass ratio: 1:1, 2:1, 3:1, 4:1) ceramics were also measured using Archimedes' technique. The relative densities were calculated from theoretical values for Zr_2_WP_2_O_12_ (3.63 g/cm^3^) and ZrO_2_ (5.817 g/cm^3^). As shown in Table 2, the results are consistent with the SEM analysis above. The relative densities of pure Zr_2_WP_2_O_12_ and ZrO_2_ were low, however, the densities of Zr_2_WP_2_O_12_/ZrO_2_ (mass ratio: 1:1, 2:1, 3:1, 4:1) ceramics increased with increasing content of Zr_2_WP_2_O_12_. For a 4:1 mass ratio Zr_2_WP_2_O_12_/ZrO_2_ composite, the relative density of the sample reached 91.5% of the theoretical density values. The sintering temperature of Zr_2_WP_2_O_12_ is lower than that of ZrO_2_, which results in a decreased sintering temperature and better densification of Zr_2_WP_2_O_12_/ZrO_2_ ceramics with increasing content of Zr_2_WP_2_O_12_.

**Table 2:** Relative densities of ZrO_2_, Zr_2_WP_2_O_12_, and Zr_2_WP_2_O_12_-ZrO_2_ composites with different mass ratios.

**Sample**	**Relative density (%)**
ZrO_2_	74.5
1:1 Zr_2_WP_2_O_12_-ZrO_2_	84.1
2:1 Zr_2_WP_2_O_12_-ZrO_2_	85.5
3:1 Zr_2_WP_2_O_12_-ZrO_2_	89.8
4:1 Zr_2_WP_2_O_12_-ZrO_2_	91.5
Zr_2_WP_2_O_12_	79.7

### Thermal expansion analysis

**Figure 4:**
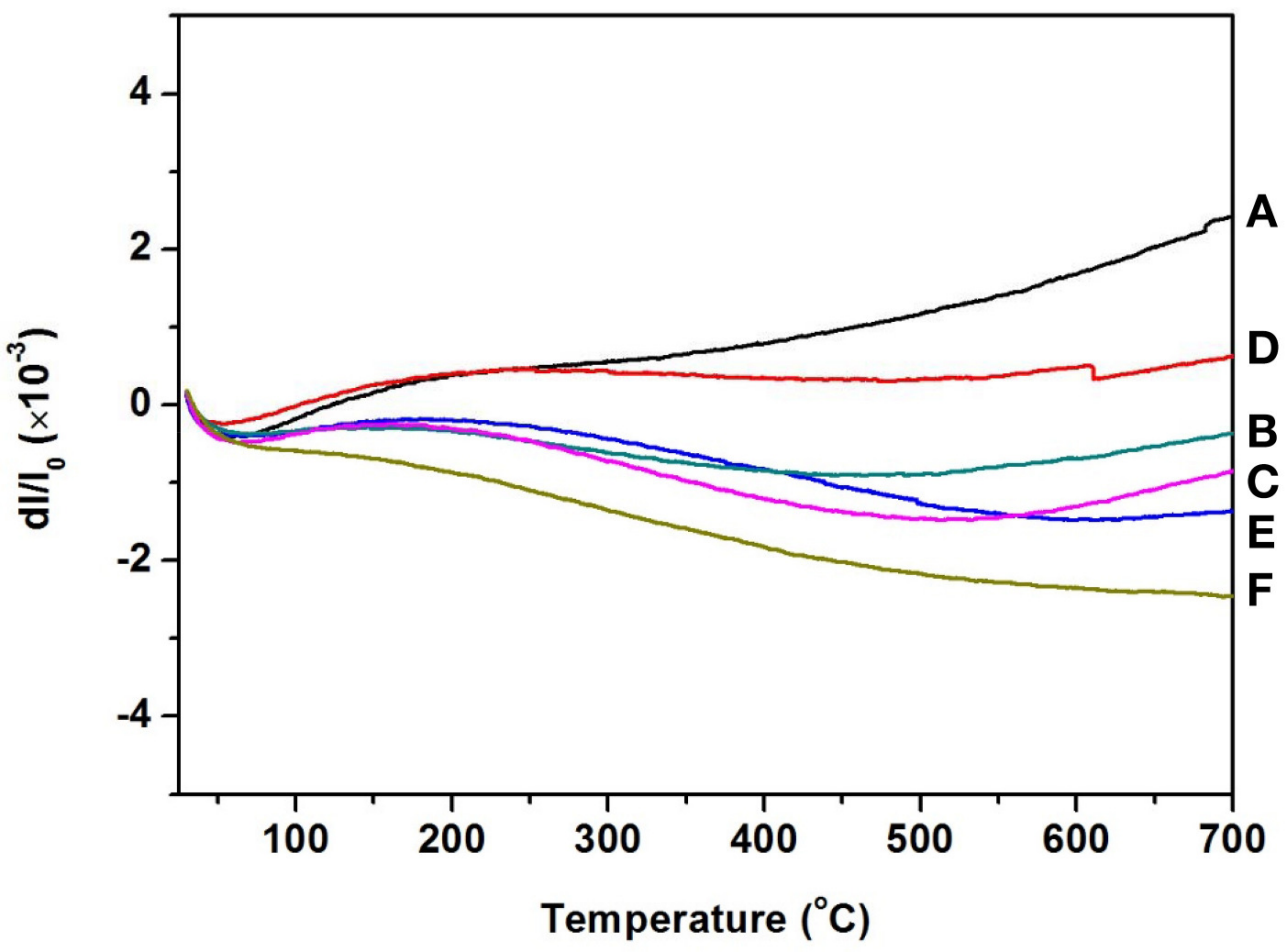
Thermal expansion curves of ZrO_2_, Zr_2_WP_2_O_12_, and Zr_2_WP_2_O_12_-ZrO_2_ composites. **(A)** ZrO_2_, **(B)** 1:1 Zr_2_WP_2_O_12_:ZrO_2_, **(C)** 2:1 Zr_2_WP_2_O_12_:ZrO_2_, **(D)** 3:1 Zr_2_WP_2_O_12_:ZrO_2_, **(E)** 4:1 Zr_2_WP_2_O_12_-ZrO_2_, **(F)** Zr_2_WP_2_O_12_.

Figure 4 gives the information about the thermal expansion of all the Zr_2_WP_2_O_12_/ZrO_2_ ceramic composites synthesized at 1,200°C for 6 h. For purposes of comparison, the thermal expansion curves of pure ZrO_2_ and pure Zr_2_WP_2_O_12_ ceramics are also given in Figure 4. Average linear CTEs of the obtained ZrO_2_, Zr_2_WP_2_O_12_, and Zr_2_WP_2_O_12_/ZrO_2_ ceramics with different mass ratios are summarized in Table 3. Pure ZrO_2_ ceramics (Figure 4A) showed positive thermal expansion between 25 and 700°C, and the average linear CTE was measured to be 4.1 × 10^−6^ K^−1^, which is lower than the value reported in the literature (Lommens et al., 2005; Yang et al., 2007). This is likely due to insufficient sintering of the ZrO_2_ ceramics, as some of the expansion can be absorbed by the empty pore space. Pure Zr_2_WP_2_O_12_ ceramics (Figure 4F) showed NTE in the testing temperature range. The average linear CTE of the Zr_2_WP_2_O_12_ ceramics was measured to be −3.3 × 10^−6^ K^−1^ in the temperature range of 25–700°C, which is consistent with literature reports (Cetinkol and Wilkinson, 2009; Isobe et al., 2009). As can be expected, the CTEs of the Zr_2_WP_2_O_12_/ZrO_2_ composites decreased from 4.1 × 10^−6^ K^−1^ to −3.3 × 10^−6^ K^−1^ as the weight fraction of Zr_2_WP_2_O_12_ was increased. As shown in Figure 4C, the 2:1 Zr_2_WP_2_O_12_/ZrO_2_ specimen showed close to zero thermal expansion with an average linear CTE of −0.09 × 10^−6^ K^−1^ in the temperature range of 25–700°C. This near zero expansion ceramic composite will have a number of potential applications in many fields. These results suggest that the CTE of the Zr_2_WP_2_O_12_-ZrO_2_ composites can be modified in the range from 4.1 × 10^−6^ K^−1^ to −3.3 × 10^−6^ K^−1^, and that it is even possible to achieve zero thermal expansion by adjusting the mass ratios of Zr_2_WP_2_O_12_ and ZrO_2_.

**Table 3:** Average linear thermal expansion coefficients of ZrO_2_, Zr_2_WP_2_O_12_, and Zr_2_WP_2_O_12_-ZrO_2_ composites in corresponding testing temperature range from 25 to 700°C.

**Samples**	**Coefficient of thermal expansion**
ZrO_2_	4.10 × 10^−6^ K^−1^
1:1 Zr_2_WP_2_O_12_-ZrO_2_	1.32 × 10^−6^ K^−1^
2:1 Zr_2_WP_2_O_12_-ZrO_2_	−0.09 × 10^−6^ K^−1^
3:1 Zr_2_WP_2_O_12_-ZrO_2_	−0.88 × 10^−6^ K^−1^
4:1 Zr_2_WP_2_O_12_-ZrO_2_	−1.50 × 10^−6^ K^−1^
Zr_2_WP_2_O_12_	−3.30 × 10^−6^ K^−1^

## Conclusions

Zr_2_WP_2_O_12_/ZrO_2_ ceramic composites with adjustable thermal expansion coefficients were successfully fabricated by a solid state reaction method. The composites consisted of orthorhombic Zr_2_WP_2_O_12_ and monoclinic ZrO_2_ with no intermediate phases observed. With increasing amount of Zr_2_WP_2_O_12_, the relative densities of the Zr_2_WP_2_O_12_/ZrO_2_ ceramic composites increased gradually. The CTE of the Zr_2_WP_2_O_12_/ZrO_2_ composites can be tailored from 4.1 × 10^−6^ K^−1^ to −3.3 × 10^−6^ K^−1^ by changing the weight fraction of Zr_2_WP_2_O_12_. For a mass ratio of Zr_2_WP_2_O_12_/ZrO_2_ of 2:1, the Zr_2_WP_2_O_12_/ZrO_2_ ceramic composite showed close to zero thermal expansion with an average linear CTE of −0.09 × 10^−6^ K^−1^ between 25 and 700°C.

## Author contributions

HL, XC, and ZZ designed experiments; WS and GX carried out experiments; HL, ZZ, and XZ analyzed experimental results and wrote the manuscript.

### Conflict of interest statement

The authors declare that the research was conducted in the absence of any commercial or financial relationships that could be construed as a potential conflict of interest.
